# Image-directed, tissue-preserving focal therapy of prostate cancer: a feasibility study of a novel deformable magnetic resonance-ultrasound (MR-US) registration system

**DOI:** 10.1111/bju.12223

**Published:** 2013-07-02

**Authors:** Louise Dickinson, Yipeng Hu, Hashim U Ahmed, Clare Allen, Alex P Kirkham, Mark Emberton, Dean Barratt

**Affiliations:** * Department of Urology, University College London Hospitals NHS Foundation Trust; § Department of Radiology, University College London Hospitals NHS Foundation Trust; † Division of Surgery and Interventional Sciences, Univeristy College LondonLondon, UK; ‡ Centre for Medical Image Computing and Department of Medical Physics and Bioengineering, Univeristy College LondonLondon, UK

**Keywords:** multi-parametric MRI, prostate cancer, focal therapy, MR-US registration

## Abstract

**Objective:**

- To evaluate the feasibility of using computer-assisted, deformable image registration software to enable three-dimensional (3D), multi-parametric (mp) magnetic resonance imaging (MRI)-derived information on tumour location and extent, to inform the planning and conduct of focal high-intensity focused ultrasound (HIFU) therapy.

**Patients and Methods:**

- A nested pilot study of 26 consecutive men with a visible discrete focus on mpMRI, correlating with positive histology on transperineal template mapping biopsy, who underwent focal HIFU (Sonablate 500®) within a prospective, Ethics Committee-approved multicentre trial (‘INDEX’).
- Non-rigid image registration software developed in our institution was used to transfer data on the location and limits of the index lesion as defined by mpMRI.
- Manual contouring of the prostate capsule and histologically confirmed MR-visible lesion was performed preoperatively by a urologist and uro-radiologist.
- A deformable patient-specific computer model, which captures the location of the target lesion, was automatically generated for each patient and registered to a 3D transrectal ultrasonography (US) volume using a small number (10–20) of manually defined capsule points.
- During the focal HIFU, the urologist could add additional sonications after image-registration if it was felt that the original treatment plan did not cover the lesion sufficiently with a margin.

**Results:**

- Prostate capsule and lesion contouring was achieved in <5 min preoperatively. The mean (range) time taken to register images was 6 (3–16) min.
- Additional treatment sonications were added in 13 of 26 cases leading to a mean (range) additional treatment time of 45 (9–90) s.

**Conclusion:**

- Non-rigid MR-US registration is feasible, efficient and can locate lesions on US.
- The process has potential for improved accuracy of focal treatments, and improved diagnostic sampling strategies for prostate cancer.
- Further work on whether deformable MR-US registration impacts on efficacy is required.

## Introduction

Tissue preserving, focal treatments for early prostate cancer are currently under early phase evaluation within prospective development studies with encouraging early risk/benefit ratio outcomes 1–3. The early proof-of-concept studies used treatment regions based on anatomical boundaries rather than the tumour, e.g. ‘hemi-ablation’ 1,3. More contemporary reports have moved towards defining targets and planned treatment volumes by cancer foci, including a treatment margin 2. This is established practice in radiation therapy, where the process of treatment planning is heavily dependent on imaging to define the gross tumour volume, clinical target volume and planning target volume 4,5.

The key challenge in tissue-preserving focal ablative methods is to enable the operator to target the tumour volume during the ultrasound (US)-based procedure (detected and defined before treatment) with an appropriate cancer margin, in a similar manner to the treatment planning procedures used in radiotherapy. The technical obstacles to this are significant in that they must account for differences between diagnostic images (often multi-parametric (mp) MRI) and those images taken on the treatment platform (often US), arising through position, rotation, compression and swelling deformation. Further, although novel MR-targeted ablative techniques are now being described within small case series 6, these are performed ‘in-bore’, which is costly, resource intensive, and requires MR-compatible equipment. Image registration aims to overcome these problems. The ability to electronically translate and transfer information on cancer burden, multiplicity and location to inform and direct therapy is currently not a standard feature of most existing ablative therapy platforms accessible to urologists.

Image-fusion technologies have now been developed for aiding MRI-targeted biopsies to suspicious areas 7–10, including several commercially available systems. However, these systems have not yet been used for targeted, tissue-preserving, therapies. Furthermore, many of these systems do not account for the deformity issues described above.

We have developed advanced, deformable, semi-automatic image registration software at our institution to achieve this. In the present study, we report on our early clinical experience, which is the first of this kind to our knowledge, in the feasibility of using image-registration to guide and deliver tissue preserving focal therapy by incorporating three-dimensional (3D) MR-visible lesion information (verified by transperineal template mapping biopsies) for decisions on ablation margins. We nested a pilot study within the ‘INDEX’ trial, a multi-centre, investigator-led, UK National Cancer Research Network study evaluating 3-year outcomes after focal high-intensity focused ultrasound (HIFU). Within the pilot study, we sought to firstly assess the efficiency of work-flows in clinically integrating image-registration into the trial pathway, and secondly, to ensure that the registered information could be visualised and resembled the information obtained on preoperative mpMRI. Thirdly, we aimed to obtain pilot data on whether MR-US registration resulted in a change in the treatment plan. As a pilot study, verification that the registration technique had any clinical utility within this trial cohort was outside of the scope of this report.

## Patients and Methods

A proof-of-concept feasibility study of image-registration was nested within an existing registered, prospective, ethics committee approved multicentre trial (‘INDEX’) of focal therapy using HIFU (Sonablate 500®) (clinicaltrials.gov NCT01194648); ‘INDEX’ meets the definition of a Prospective Exploration study for surgical research 11 and will eventually recruit 140 men with localised prostate cancer (PSA level of ≤15 ng/mL, ≤ radiological T3aN0M0, Gleason ≤ 4 + 3) who will be treated based on histological findings from a 5-mm transperineal template-guided mapping biopsy. Before treatment, all patients undergo pre-biopsy mpMRI, including T2-weighting, diffusion-weighting, and dynamic contrast-enhancement. Our nested study involved integrating computer-assisted MR-TRUS image registration software within the planning and conduct of the first 26 men treated with focal HIFU who had an MR-lesion at one participating trial centre.

The objectives were to evaluate workflows, user interaction and software to hardware integration and stability. We also aimed to obtain pilot data on the extent to which the image registration software aided our treatment planning when defining the margin around areas of MR-lesions.

### Workflow for MR-directed therapy

**Figure 1:**
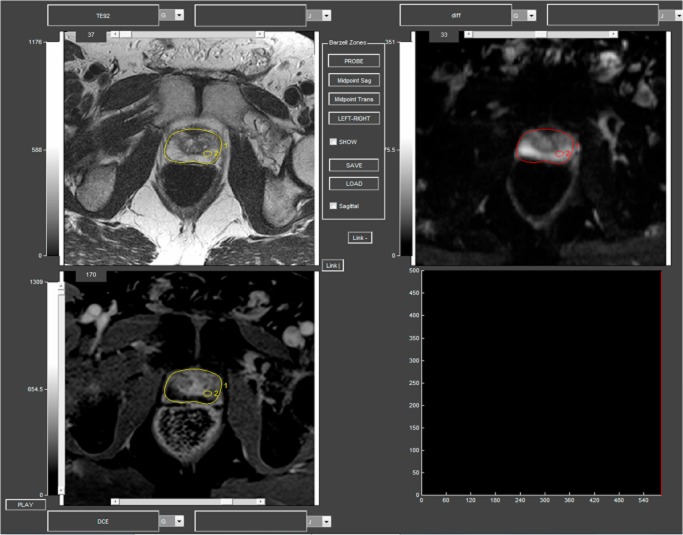
Manual contouring of the MR-visible lesion on axial T2-weighted images from a preoperative mpMRI. Dynamic contrast-enhanced and diffusion-weighted images are also displayed on the same screen. The prostatic capsule (contour 1) (apex to base) and the MR-visible lesion (contour 2) were contoured separately.

Manual contouring of the prostate capsule and the MR-visible lesion on transverse (axial) slices of the T2-weighted MR volume was performed preoperatively by a urologist (L.D.) and/or uro-radiologist (A.K., C.A.) (Fig. 1). A 3D, deformable, patient-specific computer model of the prostate and target lesion, which captures its location, shape, and size was then generated automatically for each patient using custom-written software developed by our research group 12.

An important property of these computer models is that they deform in a physically realistic way to compensate for TRUS-probe induced shape changes that occur. This is achieved by using computer simulations to predict the plausible deformation of the prostate gland during the insertion of the TRUS probe. We have previously described the full technical details 11,13.

### Therapy Planning

The focal HIFU treatment protocol for ‘INDEX’ was to treat the prostate side that had the dominant or index lesion using quadrant ablation, hemi-ablation, or hemi-ablation with contralateral extension where disease crossed the mid-line (maximum 60% gland ablation). The initial planning was based on operator judgements, primarily based on position of disease on template prostate-mapping biopsy and visual inspection of the mpMR-images on a separate workstation. This was carried out before the registration process.

### Intraoperative Image Registration

After the initial cognitive based treatment plan, intraoperative MR-TRUS registration was performed on a separate computer workstation. The following steps were required: first, acquisition of a 3D TRUS volume using the HIFU probe's imaging facility; second, a number of points were defined on both sagittal and transverse images (10–30 points) to define the prostate contour on the US 3D volume; and third, the MR-derived, patient-specific model was then registered automatically to these points and displayed as a graphical overlay on the TRUS image views, as shown in Fig. 2 11.

**Figure 2:**
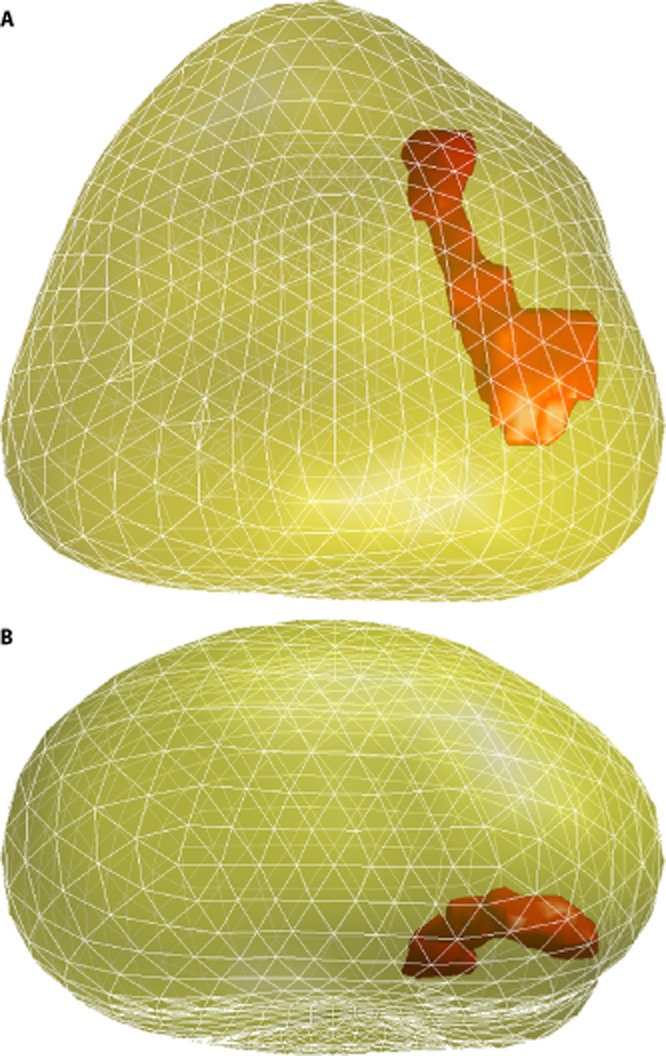
A and B. An automatically generated 3D, deformable, patient-specific computer model of the prostate and target lesion (shown in orange) using the custom-written software developed by our research group 11.

### Therapy Planning Adjustment after Target Lesion Registration

**Figure 3:**
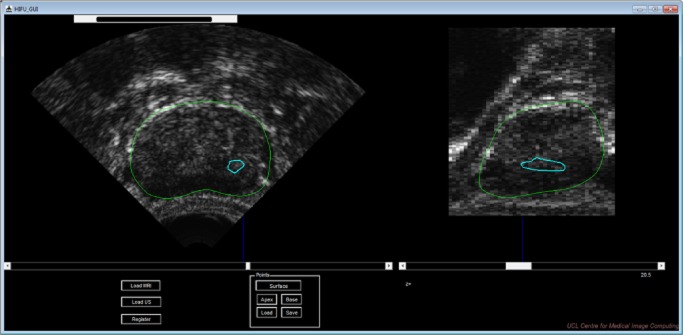
A screenshot of the image registration software: The patient-specific deformable model (green and blue) is displayed as a graphical overlay on the real-time TRUS images (taken from the HIFU device) after registration. This was preceded by manual definition of the limits of prostatic capsule in the TRUS image and the definition of 10–20 user-defined points on at least one transverse and sagittal slices.

**Figure 4:**
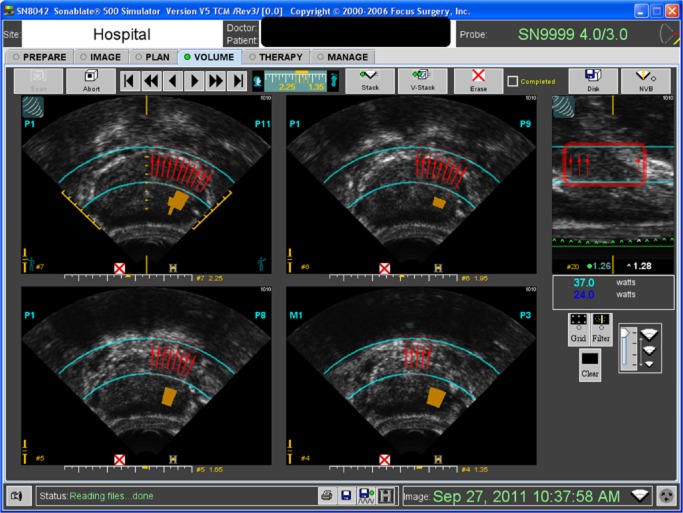
Screenshot from the HIFU therapy planning software: A visual representation of the target lesion volume is displayed as a coloured (yellow) overlay on the TRUS images on the HIFU workstation. The red lines define the region to be treated.

The target lesion volume was then displayed as a coloured overlay on TRUS images as shown in Fig. 3 and Fig. 4. As the objective was to evaluate the feasibility of using our image-registration software in the clinical setting, best ethical practice dictated that treatment volume could be added but not subtracted after image-registration. This ensured that cancer ablation was not compromised, and only (possibly) enhanced.

## Results

The clinical and tumour characteristics of the men treated are detailed in Table 1. T-stage was based on radiological stage from mpMRI as is standard practice in our cancer network. In the first 26 cases reported here, the intention was to verify that use of the image-registration process in the clinical setting would not interfere adversely with the intraoperative workflow. Information on location of the index lesion was concordant between mpMRI and template biopsy in all cases. There were no adverse events reported as a result of using the image-registration software. The HIFU device did not report any technical issues or problems when linked to the external computer workstation housing the image-registration software. The registered lesion appeared appropriately depicted on the HIFU device, correlating with expected position and volume (for the pixel size used). Prostate capsule and lesion contouring could be achieved in <5 min preoperatively. The mean (range) time taken to register images was 6 (3–16) min. This was measured from the time that the TRUS volume was transferred onto the separate workstation running the registration software to the time of visualising the MR-registered lesion on the HIFU device. The initial ablation plan was modified to treat additional tissue in 13 of 26 cases leading to a mean (range) additional treatment time of 45 (9–90) s, equating to between one and 10 additional treatment sonications, within a mean (range) overall theatre time of 141 (95–200) min. Postoperative biochemical and histological outcomes are subject to a pre-defined protocol, with reporting time-points governed by a Trial Steering Committee.

**Table 1:** Clinical and tumour characteristics of the 26 patients

Patient age, years	PSA level before HIFU, μg/mL	Gleason grade	Max. CCL, mm	No. of cores taken (TPM)	No. positive cores (TPM)	Position of index lesion (MR-US fused lesion)	T stage (radiological)	D'Amico category*	Treatment added
64	1.5	3 + 4	12	58	8	Left PZ	2a	Intermediate	No
55	3.07	3 + 4	10	83	12	Right PZ	2a	Intermediate	Yes
59	5.9	3 + 4	6	24	10	Left PZ	2a	Intermediate	No
65	10.6	3 + 4	1.5	30	1	Bilateral Anterior TZ	2c	High	Yes
57	8.1	3 + 4	9	31	5	Right PZ	3a	High	Yes
57	4.05	3 + 4	7	59	7	Left PZ	2a	Intermediate	No
67	9.74	3 + 4	7	65	4	Left PZ	2a	Intermediate	No
53	12.3	3 + 4	8	45	6	Right PZ	2a	Intermediate	No
43	8.41	3 + 4	11	56	8	Left PZ	2a	Intermediate	No
73	14.2	3 + 4	3	47	5	Right PZ	2a	Intermediate	Yes
62	7.99	3 + 4	5	64	6	Left PZ	3a	High	No
74	6.53	3 + 3	5	50	2	Right PZ	2a	Low	Yes
79	9.11	3 + 4	8	109	17	Left PZ	2c	High	Yes
67	8.13	3 + 4	8	66	9	Left PZ	2a	Intermediate	No
56	6.7	3 + 4	12	55	7	Right Anterior TZ	2a	Intermediate	Yes
65	9.53	3 + 3	9	60	11	Left PZ	2a	Low	Yes
66	8.13	3 + 3	4	51	8	Left PZ	2c	High	No
57	10.76	3 + 3	11	76	19	Right PZ	2a	Intermediate	Yes
66	10.16	3 + 4	3	49	4	Bilateral Anterior TZ	2c	High	Yes
54	6.7	3 + 3	4	32	9	Anterior TZ	2c	High	No
40	2.61	3 + 3	12	48	12	Right PZ	2a	Low	Yes
59	5.93	3 + 3	5	48	13	Right PZ	2c	High	No
63	4.54	3 + 3	5	55	11	Mid PZ	2c	High	No
54	8.07	3 + 4	7	69	14	Right PZ	2c	High	No
68	9.3	3 + 4	3	47	3	Bilateral Anterior TZ	2c	High	Yes
65	7.91	3 + 3	8	29	11	Bilateral Anterior TZ	2c	High	Yes

CCL, cancer core length; TPM, transperineal template-mapping biopsy; PZ, peripheral zone; TZ, transition zone, *Based on mp radiological stage.

## Discussion

We have shown that deformable image registration is feasible and safe when introduced into an ablative therapy setting. Moreover, most of the additional workflow and time can be carried out elsewhere with minimal time required in the operating room. Further, there is the potential for improving the accuracy of incorporating a therapeutic margin and targeting lesions within a tissue-preserving focal therapy approach.

Indeed, if on-going clinical trials show clinical utility for focal therapy within standard care, it is possible that image-registration software may be essential for the efficient implementation of truly focal therapy techniques in which individual tumours are treated within an appropriate and safe surgical margin. As shown in the present series, even when quadrant or lobe ablations are used, automated registration resulted in additional treatment volume in half of the cases. The volume added equated to up to approximately 1 mL of tissue and may have an impact on disease-free status. Within the confines of a nested pilot study, we will not be able to truly verify any disease-control advantage. There appeared to be no correlation between tumour position and the addition of treatment sonications, albeit that most patients had peripheral zone lesions. In particular, two patients had radiological T3a disease, but treatment was added in only one of these cases.

There are several commercially available software-driven devices that aim to register MRI data with US images for the purposes of prostate biopsy and therapy guidance. However, a significant limitation of many of these techniques is that, unlike the software used in the present series, changes in the size and shape of the prostate that result from differences in patient position and the insertion of a TRUS probe (and/or an endorectal MR coil) are not compensated for. More sophisticated deformable, or ‘non-rigid’, registration methods, include those that have only been validated using a phantom, and require full contouring of the prostate in TRUS images 14,15. This is time-consuming and undesirable in the intraoperative setting. Some recent series have reported good accuracy of their deformable method on phantom and clinical images, but without therapeutic application of their results 16,17. Our group has recently described a deformable image-registration method that automatically aligns an MR image to a 3D TRUS image 4, with a registration accuracy of 2.40 mm. In the present study, we used an adapted, semi-automatic version of the algorithm described in our earlier work for which a number of user-defined prostate capsule points were identified in 3–5 TRUS image slices without the need to contour the entire gland. This resulted in a system that was robust to the variable quality of different TRUS imaging systems.

There are several limitations to the present study. First, although our image-registration process takes an average time of <10 min, additional time was required to manually contour the lesion and prostatic capsule on MR-images, and to pre-process the MRI data before therapy. This timescale was not incorporated into the present results. Second, although prostate tumours are not always most clearly visualised on the T2-weighted sequence, contouring could only be performed using this sequence on the current software. However, diffusion-weighted and dynamic contrast-enhanced sequences could also be visualised within the same screen at the time of contouring for reference. Third, for these first few cases, expert knowledge from members of the radiology, urology and computing departments was required. This was anticipated and with increased use and experience, and refinement of the software interface, it is likely that the time and person burden will diminish. The learning curve is likely to mirror that of most image-based procedures. Finally, whilst we did add sonications to our original operator-defined treatment plans as a result of image-registration, within this feasibility study it was not our intention to evaluate the impact on cancer control. This will ideally require a randomised comparative approach of cognitive based treatment planning vs an automated computer based image-registration driven plan. We plan to do this once the pre-defined protocol for the first 140 men treated in the ‘INDEX’ trial is complete.

Although the clinical adoption of an MR-directed therapy is likely to add cost to existing workflows and pathways, it may provide potential for overall health economic saving in the future through improved cancer diagnosis (through image-targeted biopsy) and improved therapeutic cancer control. The use of MR-US registration potentially provides a highly cost-effective solution that, as shown in the present study, can be easily integrated within existing workflows and interfaces, with standard surgical equipment. However, we accept that formal cost analyses will be required, to further quantify this.

To the best of our knowledge, this is the first report of integrating a deformable, non-rigid, image-registration system for MR-directed HIFU therapy planning. If further evaluation shows a high level of accuracy, ease-of-use, and inter-observer reproducibility, integration of this software may allow improved accuracy of targeting and delivery of highly selective tissue treatment, with minimal burden to resources, and easy adoption by clinicians if focal therapy is shown to have a role in standard clinical practice.

## Abbreviations

3D: three-dimensional
HIFU: high-intensity focused ultrasound
mp: multi-parametric
US: ultrasound/ultrasonography
